# Genital Warts and Male Sexual Dysfunction: An IIEF-15-Based Cross-Sectional Study

**DOI:** 10.3390/healthcare14132009

**Published:** 2026-07-06

**Authors:** Orhan Şen, Emre Kıraç

**Affiliations:** 1 Department of Dermatology, Kayseri City Education and Research Hospital, Kayseri 38080, Turkey; 2 Department of Urology, Sivas Numune Hospital, Sivas 58060, Turkey; emrekrac@hotmail.com

**Keywords:** genital warts, human papillomavirus (HPV), erectile dysfunction, IIEF-15, psychosexual dysfunction

## Abstract

**Highlights:**

**What are the main findings?**

- High Prevalence of Psychosexual Burden: Clinically evident genital warts in men are associated with a remarkably high prevalence (84.7%) of erectile dysfunction, demonstrating that the disease compromises all phases of the human sexual response cycle rather than being a minor dermatological lesion.
- Disease Duration and Sexual Function: In unadjusted analysis, longer disease duration was associated with poorer sexual function; however, this association was substantially attenuated after age adjustment, indicating that the contribution of disease chronicity requires confirmation in longitudinal studies.

**What are the implications of the main findings?**

- Shift to Multidisciplinary Care: Clinical management of male patients with genital warts may need to extend beyond physical lesion destruction alone; proactive, multidisciplinary psychosexual counseling should be integrated into standard dermatology and urology practice.
- Psychological Role of Barrier Methods: Condom use may act as an “anxiety shield” that protects sexual satisfaction by mitigating partner-transmission anxiety, a hypothesis that warrants integration into clinical sexual counseling and further validation in larger trials.

**Abstract:**

**Background/Objective:** The psychosexual burden of human papillomavirus (HPV)-induced genital warts (GWs) in men remains underexplored. This study aimed to determine the prevalence of sexual dysfunction in men with GWs and to evaluate the effects of disease duration and condom use on the sexual cycle using the International Index of Erectile Function (IIEF-15). **Methods**: This single-center, cross-sectional study was conducted in the dermatology outpatient clinic of Kayseri City Education and Research Hospital between May and June 2026 and enrolled 150 sexually active male patients with clinically diagnosed, currently present genital wart lesions. To minimize reading comprehension and social desirability biases, a two-stage protocol was implemented. First, a clinician explained each IIEF-15 item face-to-face. Patients then completed the questionnaire independently and submitted it in a sealed envelope. Associations were analyzed using Spearman’s rank correlation, and group comparisons used the Mann–Whitney U and Kruskal–Wallis tests. **Results:** The median age was 30.0 years (IQR 25.0–36.0) and the median disease duration was 18.0 months (IQR 8.0–35.8). Erectile dysfunction (ED) of varying severity was detected in 84.7% (*n* = 127) of patients. In unadjusted analysis, longer disease duration was associated with lower sexual function scores; however, these associations did not remain significant after adjustment for age. As ED severity increased, all other sexual function domains declined concurrently and strongly (Spearman rho ranging from −0.679 to −0.821; *p* < 0.0001). Condom users exhibited higher total sexual function scores than non-users (median 55.5, IQR 43.2–61.0 vs. median 49.5, IQR 37.0–60.0); however, this difference did not reach statistical significance (p = 0.076). **Conclusions:** Genital warts in men constitute a major psychosexual condition that disrupts multiple phases of the sexual response cycle, not merely a dermatological lesion. Our cross-sectional findings suggest that lesion-directed treatment alone may not fully address this psychosexual burden: whether the proactive integration of multidisciplinary sexual counseling improves outcomes should be tested in controlled, longitudinal studies.

## 1. Introduction

Genital warts are predominantly caused by the low-risk HPV types 6 and 11. Although the immune system clears the infection within one to two years in the majority of cases, the virus may persist in a subset of individuals, and current ablative therapies (e.g., cryotherapy, laser, or other thermal modalities) remove visible lesions without eradicating the underlying viral infection, so recurrences are common. This combination of visible, recurrent lesions and a persistent, non-curable infection plausibly sustains the psychological burden well beyond the resolution of any single lesion [1,2,3].

Globally, the annual incidence of anogenital warts is estimated at approximately 160–289 cases per 100,000 [1]. In Turkey, where the present study was conducted, the nationwide annual prevalence of genital warts has been estimated at 154 per 100,000, with an extrapolated annual incidence of 97–131 per 100,000—figures comparable to those of European countries [4]. In Turkish cohorts, anogenital warts occur significantly more frequently in men than in women, the overwhelming majority are attributable to low-risk HPV genotypes 6 and 11, and HPV positivity is highest among men presenting with these lesions [5]. This substantial, male-predominant local burden underscores the clinical relevance of investigating the psychosexual consequences of genital warts in men in this setting [6].

The few available studies suggest that the prevalence of sexual dysfunction (SD) is higher in men with genital warts than in the healthy population, with erectile function being particularly affected [7,8]. Nevertheless, comprehensive investigations evaluating the relationship between disease chronicity and other sexual-cycle domains (sexual desire, arousal, orgasm), as well as the impact of anxiety-modulating factors such as condom use on sexual satisfaction, using multidimensional instruments, are lacking [9]. Furthermore, the literature lacks data specifically examining how sexual dysfunction resulting from genital warts is influenced by fundamental sociodemographic factors—namely educational level, marital status, and coital frequency.

Given this paucity of data in the literature, the present study is, to the best of our knowledge, among the first to evaluate sexual function in men with genital warts using a comprehensive, validated instrument such as the IIEF-15. To enhance data reliability and minimize potential reading-comprehension biases, the scale was not left to patients’ self-administration alone; instead, data were collected through face-to-face clinical interviews conducted by a specialist physician. The primary objectives of this study were: to determine the prevalence and severity of sexual dysfunction in men with genital warts; to analyze the effects of disease duration on sexual-cycle sub-domains; and to investigate the potential protective role of condom use on sexual function.

## 2. Materials and Methods

### 2.1. Study Design and Sample Size Calculation

This study was designed and reported in accordance with the Strengthening the Reporting of Observational Studies in Epidemiology (STROBE) guidelines for cross-sectional studies. The study was conducted in accordance with the principles of the Declaration of Helsinki, and ethical approval was obtained from the Ethics Committee of Kayseri City Education and Research Hospital prior to commencement (Decision No.: 922, Date: 12 May 2026). All participants were fully informed about the study objectives and the anonymization of data, and written informed consent was obtained from each patient. A blank sample of the informed consent form used in this study was provided to the editorial office to verify compliance with institutional and public transparency requirements. This single-center, cross-sectional study was conducted with 150 male patients who presented to the dermatology outpatient clinic of Kayseri City Education and Research Hospital and received a clinical diagnosis of genital warts. Inclusion criteria were: age ≥18 years, presence of clinically evident genital wart lesions, and sexual activity within the preceding four weeks. Patients with major psychiatric disorders, neurological diseases, or a history of pelvic surgery that could secondarily impair sexual function were excluded, as were those taking medications known to affect sexual function (antidepressants, antihypertensives, PDE5 inhibitors, etc.) and those with decompensated chronic disease (severe diabetes, heart failure, etc.). Sample size was determined using a priori power analysis with G*Power software (v3.1.9.7; Heinrich-Heine-Universität Düsseldorf, Düsseldorf, Germany); based on effect sizes reported in comparable sexual function studies, a minimum of 134 patients was required to detect a medium effect size (rho = 0.30) at alpha = 0.05 and power of 0.95. Enrolling 150 patients exceeded this target.

### 2.2. Data Collection Instruments and Procedure

Two instruments were used for data collection: a structured Clinical Data Form assessing sociodemographic and clinical characteristics (age, marital status, educational level, weekly coital frequency, condom use, and time elapsed since wart diagnosis), and the International Index of Erectile Function (IIEF-15) to evaluate sexual function.

Sexual function was measured using the IIEF-15, a comprehensive self-report questionnaire in which respondents rate 15 items on a Likert-type scale of 0–5 or 1–5 points, examining five key domains of the sexual cycle: erectile function, orgasmic function, sexual desire, intercourse satisfaction, and overall satisfaction. Higher scores indicate better sexual function, and the maximum achievable total score is 75. Erectile function severity was classified according to the domain subscale score as follows: 0–10 points, severe ED; 11–16 points, moderate ED; 17–21 points, mild-to-moderate ED; 22–25 points, mild ED; and 26–30 points, normal function (no ED) [10].

To maximize data reliability, a hybrid administration approach was adopted. To prevent the reading and literacy problems frequently encountered in sexual function research, the medical content and meaning of each IIEF-15 item were explained to the patient in detail by the specialist physician in the outpatient clinic setting. However, to eliminate the social desirability bias that may arise when patients are reluctant to verbalize intimate sexual problems to a clinician, patients were not asked to respond verbally. Instead, once they had fully grasped each item, patients completed the questionnaire independently—without clinician oversight—and submitted it in a sealed envelope. This strategy simultaneously ensured accurate comprehension, response honesty, and patient privacy, thereby maximizing the clinical validity of the data. The IIEF-15 was administered during the same clinical examination at which the diagnosis was made, and questionnaire completion did not delay the initiation of any treatment.

Following ethics committee approval, participant recruitment and data collection were conducted between 13 May 2026 and 5 June 2026, strictly within the period following formal ethical authorization.

### 2.3. Statistical Analysis

Statistical analysis was performed using IBM SPSS Statistics version 26.0 (IBM Corp., Armonk, NY, USA). Normality of variables was examined using the Shapiro–Wilk test, skewness-kurtosis values, and visual methods (histograms and Q-Q plots). Since the continuous variables were not normally distributed, descriptive statistics are expressed as median and interquartile range (IQR) for continuous variables, and frequency (*n*) and percentage (%) for categorical variables. Non-parametric tests were used throughout: the Mann–Whitney U test for two-group comparisons (e.g., condom use, marital status) and the Kruskal–Wallis test for comparisons involving three or more groups (e.g., educational level, weekly coital frequency). Associations between continuous variables (disease duration and sexual function scores) and between ordinal categories (ED severity) and scores were assessed using Spearman’s rank correlation coefficient. To account for the potential confounding effect of age, partial Spearman correlations between disease duration and sexual function scores were additionally computed controlling for patient age. Potential interaction effects (marital status × condom use; weekly coital frequency × condom use) on total sexual function were explored using a rank-transformed (non-parametric) two-way analysis of variance, with results reported in Supplemental Table S1. A two-tailed *p*-value < 0.05 was considered statistically significant.

### 2.4. Data Management and Variable Definitions

All data were recorded prospectively on a structured Clinical Data Form by the supervising clinician and cross-checked against the patient’s medical record. Variables were defined as follows. Age was recorded in completed years. Educational level was categorized as primary school or below, high school, or university or above. Marital status was classified as married or single with a partner. Disease duration was defined as the time, in months, elapsed since the clinical diagnosis of genital warts. Weekly coital frequency was categorized as once, twice, or three or more times per week. Condom use was defined as consistent use during every act of sexual intercourse, with any inconsistent or occasional use classified as non-use. Erectile dysfunction severity was classified according to the IIEF-15 erectile function domain subscale (0–10, severe; 11–16, moderate; 17–21, mild-to-moderate; 22–25, mild; 26–30, no ED). As data were collected under a structured, clinician-supervised administration protocol, all questionnaire items were fully completed; no missing or incomplete data were present, and therefore all 150 enrolled patients were included in the analysis.

## 3. Results

### 3.1. Sociodemographic and Clinical Characteristics

A total of 150 male patients meeting the inclusion criteria were enrolled, all of whom were included in the final analysis with no missing data. The median age was 30.0 years (IQR 25.0–36.0; range 18–48). Among the participants, 52.0% (*n* = 78) had attained university-level education or above, and 51.3% (*n* = 77) were married. Regarding clinical characteristics, the median disease duration since wart diagnosis was 18.0 months (IQR 8.0–35.8; range 1–80 months). Weekly coital frequency was ≥3 times in 40.0% (*n* = 60) of patients, and 56.0% (*n* = 84) reported not using condoms during sexual intercourse. Detailed sociodemographic and clinical data are summarized in Table 1.

### 3.2. Sexual Function Status and Erectile Dysfunction (ED) Prevalence

Because the IIEF-15 provides a validated categorical severity classification only for the erectile function domain, prevalence was reported specifically for erectile dysfunction, whereas the remaining domains of sexual function were evaluated dimensionally as continuous scores. The median total IIEF-15 score was 53.0 (IQR 40.0–61.0). Domain analysis revealed a median erectile function score of 21.0 (IQR 18.0–25.0). According to the IIEF-15 erectile function categorization, only 15.3% (*n* = 23) of patients had normal erectile function, whereas the remaining 84.7% (*n* = 127) exhibited ED of varying severity. The most common ED severity category was mild ED (33.3%). Sexual function domain scores and the distribution of ED severity are presented in Table 2.

### 3.3. Comparative and Correlation Analyses of Factors Affecting Sexual Function

Examining the association between sociodemographic variables and total IIEF score, patients with primary school or lower education had significantly lower total sexual function scores (median 41.5, IQR 27.8–50.5) than those who had completed high school (median 55.5, IQR 45.0–61.0) or university (median 53.0, IQR 37.2–61.0) (Kruskal–Wallis H = 6.540, *p* = 0.038). Patients with a weekly coital frequency of ≥3 times had significantly higher total sexual function scores (median 56.0, IQR 45.5–62.0) than those reporting once-weekly intercourse (median 44.0, IQR 32.0–56.5) (Kruskal–Wallis H = 11.702, *p* = 0.003). No significant difference in total IIEF score was observed according to marital status (median 51.0 vs. 54.0; Mann–Whitney U test, *p* = 0.302). Patients who used condoms during intercourse had higher total sexual function scores (median 55.5, IQR 43.2–61.0) than non-users (median 49.5, IQR 37.0–60.0), although this difference did not reach statistical significance (Mann–Whitney U test, *p* = 0.076).

**Figure 1:**
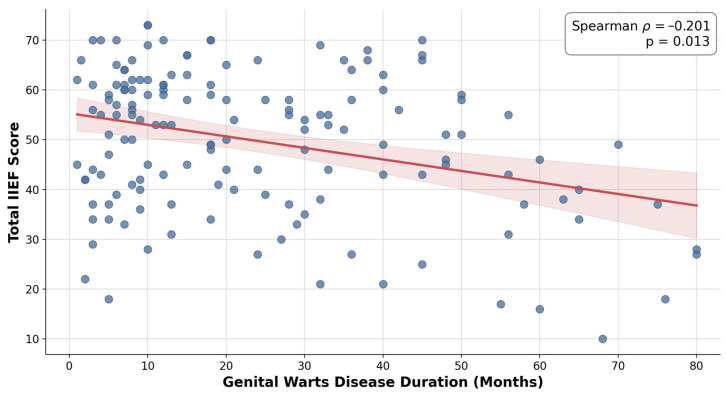
Correlation between disease duration and total sexual function score. The scatter plot illustrates the negative association between time elapsed since the clinical diagnosis of genital warts (in months) and patients’ total IIEF score, with a fitted trendline. (Spearman’s rank correlation coefficient rho = −0.201, *p* = 0.013).

As disease duration increased, statistically significant decreases were observed in total IIEF score (Spearman rho = −0.201, *p* = 0.013), erectile function (rho = −0.189, *p* = 0.021), orgasmic function (rho = −0.182, *p* = 0.026), and overall satisfaction (rho = −0.213, *p* = 0.009). In contrast, the associations of disease duration with sexual desire (rho = −0.142, *p* = 0.082) and intercourse satisfaction (rho = −0.150, *p* = 0.068) did not reach statistical significance (Figure 1).

However, because patient age was inversely associated with sexual function (age vs. total IIEF: Spearman rho = −0.365, *p* < 0.001) and positively associated with disease duration (rho = 0.178, *p* = 0.029), partial correlations controlling for age were computed. After adjustment for age, the associations between disease duration and sexual function scores were attenuated and no longer reached statistical significance for any domain (total IIEF: partial rho = −0.149, *p* = 0.070; erectile function: rho = −0.158, *p* = 0.055; orgasmic function: rho = −0.129, *p* = 0.116; overall satisfaction: rho = −0.154, *p* = 0.061). These findings indicate that the apparent association between longer disease duration and poorer sexual function is substantially confounded by age and should be interpreted as hypothesis-generating rather than confirmatory.

**Figure 2:**
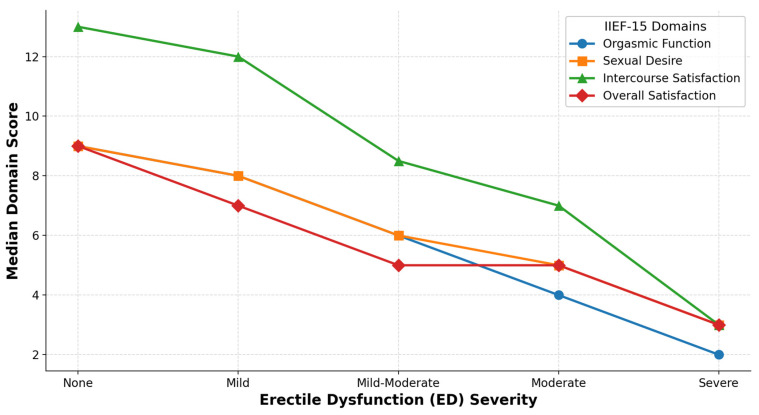
The effect of erectile dysfunction severity on other domains of sexual function. This line graph demonstrates the concurrent and dramatic decline (domino effect) in other mechanisms of sexual function (orgasmic function, sexual desire, intercourse satisfaction, and overall satisfaction) as the severity of erectile dysfunction (ED) increases (None, Mild, Mild–Moderate, Moderate, Severe). The ED categories are ordinally ranked, and each data point represents the median score of the respective domain for the patients in that group. (Spearman’s rank correlation, *p* < 0.0001 for all domains).

In addition, the impact of ED severity on other sexual function domains was examined using Spearman’s rank correlation. As ED severity increased, statistically significant (*p* < 0.0001) and strong deterioration was observed in orgasmic function (rho = −0.821), sexual desire (rho = −0.706), intercourse satisfaction (rho = −0.680), and overall satisfaction (rho = −0.679) (Table 3, Figure 2).

Exploratory interaction analyses showed no significant interaction between condom use and either marital status (*p* = 0.530) or weekly coital frequency (*p* = 0.732) on total sexual function, indicating that the association of condom use with sexual function did not differ across these subgroups (Supplemental Table S1).

## 4. Discussion

This study is among the largest and most methodologically rigorous investigations evaluating the prevalence and severity of sexual dysfunction in men with genital warts (GWs). Our findings demonstrate that 84.7% of men with genital warts exhibit ED of varying severity, and that the sexual cycle (desire, arousal, orgasm) is secondarily affected as the disease progresses. The detrimental effects of other sexually transmitted infections—particularly HIV and HSV—on sexual function are well established in the literature [11,12]. However, the specific detrimental impact of HPV-induced genital warts on male sexual health has been largely overlooked. The present study demonstrates that genital warts are not merely a cosmetic or dermatological lesion but a major sexual health problem that threatens the patient’s psychosexual integrity [13].

The ED rate of 84.7% identified in our study substantially exceeds the ED prevalence reported in the general healthy population—where erectile dysfunction affects only an estimated 5–10% of men under 40 years of age—indicating the considerable anxiety burden imposed by the disease [14]. This finding is consistent with the 85.7% erectile impairment rate reported by Nia et al. among 105 male patients with genital warts [7]. Notably, whereas patients with age-related organic ED typically retain sexual desire, our findings reveal a severe decline in sexual desire, orgasmic function, and overall satisfaction as ED severity increases (*p* < 0.0001) [15]. This distinction suggests that the sexual dysfunction observed in patients with genital warts is predominantly psychogenic in origin—driven by loss of self-confidence, stigmatization, disturbed body image, and fear of transmitting the infection to partners—rather than being attributable to vascular or neurological mechanisms [8].

The literature to date has largely focused on the acute psychological impact of the disease at the time of diagnosis. Our data demonstrate that as the time elapsed since wart diagnosis increases, patients’ total IIEF, erectile function, orgasmic function, and overall satisfaction scores decline significantly (Spearman rho = −0.201 for total IIEF, *p* = 0.013), whereas the associations with sexual desire and intercourse satisfaction did not reach significance. Importantly, however, when patient age was statistically controlled, these associations were attenuated and no longer reached significance, indicating that the apparent relationship between disease chronicity and sexual dysfunction is substantially confounded by age. This pattern should therefore be regarded as hypothesis-generating: although disease chronicity, repeated treatment procedures, and prolonged transmission risk may plausibly contribute to persistent sexual avoidance and performance anxiety, longitudinal studies are required to establish whether duration exerts an independent effect.

Our study also yielded compelling findings regarding the influence of sociodemographic factors on sexual function in men with genital warts. Higher educational level and greater weekly coital frequency were both associated with statistically significantly higher sexual function scores (*p* < 0.05). The protective effect of higher education may be explained by greater health literacy, better comprehension of the nature of HPV infection, and an enhanced ability to overcome maladaptive illness cognitions [16]. Perhaps the most striking finding of our study is that marital status had no statistically significant effect on the severity of sexual dysfunction (*p* = 0.302). Although marriage and a stable partnership are generally considered protective factors for sexual function in the general population, the loss of this protective effect in a genital warts cohort is particularly noteworthy [17]. This observation implies that the fear of transmitting the infection and bodily shame generated by genital warts may contribute to a pervasive psychosexual burden and performance anxiety across affected men, regardless of marital bond or partnership stability. The finding that patients with lower coital frequency had lower IIEF scores points to a bidirectional interaction: patients avoid intercourse out of shame regarding their lesions or fear of transmission, and diminished sexual contact secondarily further impairs erectile function [18].

Another striking and novel contribution of this study relates to the effect of condom use on sexual function. Our analyses reveal that patients who used condoms during intercourse had higher total sexual function scores than non-users, a difference that did not reach statistical significance (Mann–Whitney U test, *p* = 0.076). Although condom use is clinically assumed to reduce penile sensitivity and thus negatively affect erection, our data in a genital warts cohort suggest the hypothesis that the condom may serve as a psychological shield [19]. Notably, because condoms do not provide complete protection against HPV transmission—the virus can infect basal keratinocytes via syndecan-1 and other receptors at sites not covered by the condom—any benefit observed here is more plausibly attributable to a reduction in transmission-related anxiety than to a true reduction in transmission risk, which reinforces rather than undermines the proposed psychological mechanism [20]. By reducing the fear of transmitting the virus to their partner, condom-using patients appear to suppress performance anxiety, thereby preserving erectile function and sexual satisfaction. This finding indicates that, in the sexual counseling of GW patients, barrier methods may be considered not only for infection prevention but also for the purpose of protecting sexual function [21].

The study possesses several important methodological strengths that enhance the validity of its findings. In sexual function research, data collection is typically conducted through online surveys or unmonitored self-administered forms, which may lead to misinterpretation of questions. Conversely, traditional interviews in which the clinician poses questions and records answers directly may result in patients concealing intimate problems out of embarrassment. The present study employed a hybrid approach that addresses both major sources of bias: a specialist physician explained the medical content and scope of each IIEF-15 item face-to-face in the outpatient setting, while the form was completed independently by the patient with full privacy ensured. This proactive strategy minimized comprehension errors attributable to educational level and enabled patients to provide candid responses, thereby maximizing the clinical validity of the data.

Several limitations of this study warrant consideration. The cross-sectional design precludes definitive causal inference regarding the relationship between disease duration and erectile dysfunction. As a single-center study conducted at a single tertiary hospital, its findings may have limited generalizability. The study population also consisted predominantly of young and middle-aged men (median age 30.0 years), and the results may therefore not extend to older age groups, in whom organic and vascular contributors to erectile dysfunction are more prevalent. The absence of a healthy control group further precludes direct comparison with the general population, and depression and anxiety levels—which may confound sexual function—were not measured using a specific psychometric instrument (e.g., Beck Depression Inventory or HADS). In addition, certain baseline characteristics that may influence erectile function, such as body mass index, smoking status, alcohol consumption, and prior HPV vaccination, were not recorded, as the present study focused specifically on the psychosexual impact of genital warts. The localization and lesion burden of genital warts were also not systematically recorded, which may limit analysis of morphology-related effects. Finally, sexual function and behavior were assessed using a self-reported instrument (IIEF-15), which may be subject to recall and reporting biases. Future studies incorporating a healthy control group and concurrently examining the impact of male patients’ sexual dysfunction on the sexual function of female partners would represent a substantial contribution to the literature. In particular, prospective longitudinal designs are needed to clarify whether erectile and overall sexual function recover following complete or partial clearance of genital warts, how long such recovery may take, and whether earlier or more rapid lesion treatment is associated with improved sexual-function outcomes.

## 5. Conclusions

Genital warts in men are not merely a dermatological problem but a major psychosexual condition that affects all phases of the sexual cycle, most prominently erectile function. Longer disease duration was associated with poorer sexual function, although this relationship was substantially attenuated after adjustment for age and should therefore be regarded as hypothesis-generating rather than established. In dermatology and urology practice, the management of patients with genital warts should extend beyond lesion destruction; the psychosexual status and partner relationships of affected men should be routinely evaluated through a proactive, multidisciplinary approach. From a public health perspective, three measures merit emphasis: first, the integration of brief psychosexual screening into the routine care of patients with genital warts; second, the strengthening of structured patient education and counseling to address transmission-related anxiety and stigma; third, the promotion of universal HPV vaccination, which offers the most effective primary-prevention strategy against HPV-related disease and its associated psychosexual burden.

## Figures and Tables

**Table 1:** Sociodemographic and Clinical Characteristics of Patients (*n* = 150).

Variables	*n* (%)/Median (IQR)
Age (years)	30.0 (25.0–36.0)
**Education level**	
Primary school or below	18 (12.0%)
High school	54 (36.0%)
University or above	78 (52.0%)
**Marital status**	
Married	77 (51.3%)
Single (with partner)	73 (48.7%)
**Weekly sexual intercourse frequency**	
Once	47 (31.3%)
Twice	43 (28.7%)
≥3 times	60 (40.0%)
**Condom use**	
Yes	66 (44.0%)
No	84 (56.0%)
Disease duration (months)	18.0 (8.0–35.8)

Continuous variables are presented as median (interquartile range, IQR); categorical variables as *n* (%). IQR = interquartile range.

**Table 2:** Sexual Function Domain Scores and Distribution of ED Severity.

IIEF-15 Domains	Median (IQR)	Minimum–Maximum
Erectile Function (EF)	21.0 (18.0–25.0)	1.0–30.0
Orgasmic Function	7.0 (5.0–9.0)	0–10.0
Sexual Desire	7.0 (5.0–8.0)	2.0–10.0
Intercourse Satisfaction	10.0 (7.0–12.0)	1.0–15.0
Overall Satisfaction	7.0 (5.0–8.0)	2.0–10.0
Total IIEF Score	53.0 (40.0–61.0)	10.0–73.0
**ED severity categories**	***n***	**Percentage (%)**
None (healthy, ≥26)	23	15.3
Mild (22–25)	50	33.3
Mild-to-moderate (17–21)	48	32.0
Moderate (11–16)	19	12.7
Severe (≤10)	10	6.7

Domain scores are presented as median (IQR). ED = erectile dysfunction; IIEF-15 = International Index of Erectile Function.

**Table 3:** Spearman Correlations of Disease Duration (crude and age-adjusted) and ED Severity with Sexual Function Domains.

Sexual Function Domain	Disease Duration, Crude (rho)	*p*	Disease Duration, Age-Adjusted (Partial rho)	*p*	ED Severity (rho) *
Erectile Function (EF)	−0.189	0.021	−0.158	0.055	—
Orgasmic Function	−0.182	0.026	−0.129	0.116	−0.821
Sexual Desire	−0.142	0.082	−0.068	0.413	−0.706
Intercourse Satisfaction	−0.150	0.068	−0.093	0.260	−0.680
Overall Satisfaction	−0.213	0.009	−0.154	0.061	−0.679
Total IIEF Score	−0.201	0.013	−0.149	0.070	—

All correlations are Spearman’s rank correlation coefficients (rho). Crude = unadjusted; age-adjusted = partial correlation controlling for patient age. ED severity was coded ordinally (None = 0, Mild = 1, Mild-to-moderate = 2, Moderate = 3, Severe = 4); all ED-severity correlations were significant at *p* < 0.0001 (*). ED = erectile dysfunction.

## Data Availability

The data presented in this study are available on request from the corresponding author due to privacy and ethical restrictions. The data are not publicly available to ensure patient confidentiality.

## Supplementary Materials

The following supporting information can be downloaded at: https://www.mdpi.com/article/10.3390/healthcare14132009/s1. Table S1: Two-way (rank-transformed) interaction analyses for total IIEF score (*n* = 150).

## Abbreviations

The following abbreviations are used in this manuscript:

HPV	Human papillomavirus
GWs	Genital warts
ED	Erectile dysfunction
SD	Sexual dysfunction
IIEF-15	International Index of Erectile Function
STIs	Sexually transmitted infections
HIV	Human immunodeficiency virus
HSV	Herpes simplex virus
IQR	Interquartile range
IBM SPSS	International Business Machines Statistical Package for the Social Sciences
