# Supplementary material for: Genital Warts and Male Sexual Dysfunction: An IIEF-15-Based Cross-Sectional Study
Source: Healthcare (Basel). 2026 Jul 6;14(13):2009. doi: 10.3390/healthcare14132009 (PMC13361407; doi:10.3390/healthcare14132009)
Supplement: Supplementary file 1 [file healthcare-14-02009-s001.zip › healthcare-4397705-supplementary.pdf]

Supplementary Materials

Genital Warts and Male Sexual Dysfunction: An IIEF-15-Based Cross-Sectional Study

Table S1. Two-way (rank-transformed) interaction analyses for total IIEF score (n = 150).

(a) Median total IIEF score by subgroup

Subgroup (marital status × condom use)	Total IIEF score, median
Single (with partner), condom user	56
Single (with partner), non-user	50
Married, condom user	53.5
Married, non-user	49

(b) Interaction test results

Interaction term	F	p-value	Result
Marital status × condom use	0.396	0.53	Not significant
Coital frequency × condom use	0.313	0.732	Not significant

Interactions were tested using a rank-transformed (non-parametric) two-way analysis of variance on total IIEF score (n = 150). Neither interaction term was statistically significant, indicating that the association of condom use with total sexual function did not differ across marital-status or coital-frequency subgroups. As expected, the main effect of coital frequency remained significant ($F = 5.238$, $p = 0.006$). IIEF = International Index of Erectile Function.
